# Efficacy and safety of immunosuppressive therapy combined with eltrombopag for severe aplastic anemia: a systematic review and meta-analysis

**DOI:** 10.1186/s13643-024-02515-2

**Published:** 2024-04-04

**Authors:** Yan Zhang, Jie Li, Xi Li, Qianshuang Geng, Yuqin Xie, Guoxiang Zhang, Mingxia Wei, Yanmei Ma

**Affiliations:** 1 Department of Hematology, Heji Hospital Affiliated to Changzhi Medical College, Changzhi, 046000 Shanxi China; 2 Department of Oncology and Hematology, Liuyang Hospital of Traditional Chinese Medicine, Hunan University of Chinese Medicine, Changsha, 410300 Hunan China; 3 Department of Nephrology, Heping Hospital Affiliated to Changzhi Medical College, Changzhi, 046000 Shanxi China; 4 Department of Hematology, Heping Hospital Affiliated to Changzhi Medical College, Changzhi, 046000 Shanxi China

## Abstract

**Background and objective:**

Immunosuppressive therapy (IST) is the first choice for severe aplastic anemia (SAA) patients with hematopoietic stem cell transplantation (HSCT) limitation, and the main factor limiting its efficacy is too few residual hematopoietic stem/progenitor cells (HSPC). Eltrombopag (EPAG), as a small molecule thrombopoietin receptor agonist, can stimulate the proliferation of residual HSPC and restore the bone marrow hematopoietic function of patients. In recent years, many studies have observed the efficacy and safety of IST combined with EPAG in the treatment of SAA, but the results are still controversial. The aim of this study is to systematically evaluate the efficacy and safety of IST combined with or without EPGA in the treatment of SAA.

**Methods:**

We conducted a systematic review of all relevant literature published up to January 19, 2024. Pooled odds ratio (OR) was calculated to compare the rates, along with 95% confidence intervals (CI) and *p* value to assess whether the results were statistically significant by Review Manager 5.4.1. The *p* values for the interactions between each subgroup were calculated by Stata 15.1. The Newcastle-Ottawa Scale and the Cochrane bias risk assessment tools were respectively used to evaluate the quality of the literature with cohort studies and randomized controlled trials. The Review Manager 5.4.1 and Stata 15.1 were used to assess bias risk and perform the meta-analysis.

**Results:**

A total of 16 studies involving 2148 patients were included. The IST combined with the EPAG group had higher overall response rate (ORR) than the IST group at 3 months (pooled OR = 2.10, 95% CI 1.58–2.79, *p* < 0.00001) and 6 months (pooled OR = 2.13, 95% CI 1.60–2.83, *p* < 0.00001), but the difference between the two groups became statistically insignificant at 12 months (pooled OR = 1.13, 95% CI 0.75–1.72, *p* = 0.55). The results of complete response rate (CRR) (pooled OR at 3 months = 2.73, 95% CI 1.83–4.09, *p* < 0.00001, 6 months = 2.76, 95% CI 2.08–3.67, *p* < 0.00001 and 12 months = 1.38, 95% CI 0.85–2.23, *p* = 0.19) were similar to ORR. Compared with the IST group, the IST combined with the EPAG group had better overall survival rate (OSR) (pooled OR = 1.70, 95% CI 1.15–2.51, *p* = 0.008), but there were no statistically significant differences in event-free survival rate (EFSR) (pooled OR = 1.40, 95% CI 0.93–2.13, *p* = 0.11), clonal evolution rate (pooled OR = 0.68, 95% CI 0.46–1.00, *p* = 0.05) and other adverse events between the two groups. The results of subgroup analysis showed that different ages were a source of heterogeneity, but different study types and different follow-up times were not. Moreover, all *p*-values for the interactions were greater than 0.05, suggesting that the treatment effect was not influenced by subgroup characteristics.

**Conclusion:**

EPAG added to IST enables patients to achieve earlier and faster hematologic responses with a higher rate of complete response. Although it had no effect on overall EFSR, it improved OSR and did not increase the incidence of clonal evolution and other adverse events.

**Supplementary Information:**

The online version contains supplementary material available at 10.1186/s13643-024-02515-2.

## Key points

This paper aims to perform a meta-analysis analyzing the efficacy and safety of eltrombopag for severe aplastic anemia.

Our study suggests that EPAG added to IST is beneficial for patients with SAA.

**Supplementary Information:**

The online version contains supplementary material available at 10.1186/s13643-024-02515-2.

## Introduction

Aplastic anemia (AA) is a bone marrow hematopoietic failure syndrome mediated by abnormally activated T cells, which is often clinically manifested as anemia, bleeding, and infection [1, 2]. It is classified as severe and non-severe AA according to the severity of the disease. Severe Aplastic anemia (SAA) has acute onset, rapid progression, and high mortality. The treatment mainly includes hematopoietic stem cell transplantation (HSCT) and immunosuppressive therapy (IST) [3, 4]. Because HSCT is affected by age, donor restrictions, and other reasons, IST has become a first-line therapy for more patients who are not suitable for HSCT [5]. This protocol mainly includes anti-thymocyte globulin (ATG) and cyclosporin A (CsA), with a hematologic response rate of 60–70%, of which about 10–40% will experience relapse and about 20% will show clonal evolution [6–9]. However, clinical findings showed that after IST treatment, patients had slow bone marrow hematopoietic function recovery, excessive use of blood products, heavy economic burden, and increased risk of iron overload and organ damage [10, 11]. Several "optimized" immunosuppressive regimens have emerged in recent years, including adding a third immunosuppressive agent such as mycophenolate mofetil or sirolimus to the standard ATG + CsA two-drug regimen or replacing horse ATG with more immunosuppressive rabbit ATG or Alemtuzumab or high-dose cyclophosphamide, which have failed to improve hematologic response rates [12–17]. The most important reason is that there are too few residual HSPC in the bone marrow. Studies have shown that the lower the number of residual hematopoietic cells in a patient's bone marrow, the lower the success rate of his treatment [18].

Eltrombopag (EPAG) is an oral small molecule non-peptide thrombopoietin receptor agonist (TPO-RA), originally developed for the treatment of immune thrombocytopenia [19–21]. Because it can noncompetitively bind to the transmembrane domain of TPO-R on hematopoietic stem and progenitor cells (HSPCs), it can promote their proliferation and differentiation. At the same time, it also has functions such as regulating immunity, inducing immune tolerance, and chelating iron, so it is highly suitable for SAA [19, 22, 23].

A number of clinical studies have observed and compared the efficacy and safety of IST combined with EPAG in transplant-restricted SAA patients, but the results are still controversial. While Lesmana et al.’s study considered no effect, Hu’s study reported beneficial effects. In addition, Zaimoku et al. found that no matter what degree of SAA, IST combined with EPAG could benefit, and patients with more severe hematopoietic failure would benefit more in terms of prognosis [8].

To further elaborate on these issues, our study conducted a systematic review and meta-analysis of the studies on the treatment of SAA patients with IST combined with EPAG, in order to provide evidence-based medicine for the clinical application of EPAG.

## Materials and methods

This study was registered in the International Prospective Register of Systematic Review (PROSPERO) [CRD42023465584] and performed according to the Preferred Reporting Items for Systematic Reviews and Meta-Analyses (PRISMA) reporting guideline in 2020 [24].

### Information sources and search strategy

Two researchers (Y.Z. and J.L.) independently searched PubMed, Web of Science, Cochrane Library, Embase, China National Knowledge Infrastructure (CNKI), SinoMed, Wanfang, Vip, Chinese Clinical Trial Registry (ChiCTR), and Clinical trials from the establishment of the database to January 19, 2024. A combination of “Aplastic Anemia” and “Eltrombopag” was used as the search term to retrieve relevant studies. Moreover, we manually searched the references of all included studies to identify any other relevant studies. Full details of the search strategy for all databases are outlined in the Supplementary file.

### Inclusion and exclusion criteria

Studies published in English or Chinese meeting the following criteria were included:

#### Participants

Studies that aimed to investigate patients with SAA who were diagnosed according to the World Health Organization diagnostic criteria will be included.

#### Intervention/exposure

The studies, which use IST with EPAG for the treatment of SAA are included in this study.

#### Comparisons

The studies, which use IST for the treatment of SAA are included in this study.

#### Outcomes

The primary outcome was overall response rate (ORR) and complete response rate (CRR). Secondary outcomes included overall survival rate (OSR), event-free survival rate (EFSR) and clonal evolution.

The overall response was defined as a complete response or partial response. Complete response was defined as hemoglobin ≥ 100 g/L, platelet count ≥ 100 × 10^9^/L, ANC ≥ 1 × 10^9^/L, and the patient was no longer dependent on blood product and growth factor infusions. Partial response was defined as a blood cell count that no longer met any 2 of the 3 SAA Camitta criteria: absolute reticulocyte count (ARC) ≥ 60 × 10^9^/L, platelet count ≥ 20 × 10^9^/L, absolute neutrophil count (ANC) ≥ 0.5 × 10^9^/L. Overall survival (OS) was measured from the first day of IST until death from any cause or the date of the last follow-up. Event-free survival (EFS) was measured from the first day of IST until any event (lack of response at 6 months, HSCT, death, relapse, repeat IST, or any additional SAA treatment, transformation to PNH, and malignant clonal evolution) or the date of the last follow-up. The target endpoint was defined as the end of follow-up. Clonal evolution is defined as the appearance of cytogenetic abnormalities or characteristic changes in the bone marrow consistent with myelodysplastic syndrome (MDS) or acute myeloid leukemia (AML). The target endpoint was defined as the end of the follow-up.

#### Study design

Observational studies including (prospective and retrospective) cohort studies and randomized controlled trials (RCTs) were evaluated.

Studies were excluded if they met any of the following criteria: (1) duplicate publications; (2) meta-analysis, literature review, conference abstracts, case reports, letters, etc.; (3) The intervention was not clear, or other types of TPO-RA preparations were used; (4) studies without a control group were excluded; (5) outcome indicators with incomplete or unusable research data.

If there is any objection, the third researcher will help resolve it.

### Study selection

After searching, the records were imported into Zotero software (version 6.0) and duplicates will be removed. Then, two independent researchers (Y. Z. and J. L.) screened titles/abstracts and assessed potential full texts. Those studies fulfilling our eligibility criteria will be included in the review. If there is any objection, the third researcher will help solve it.

### Data extraction

Two researchers (Y.Z. and J.L.) independently read the included literature, extracted data independently by using a pre-specified Excel table, and cross-checked the data. If there is any objection, the third researcher will help solve it. We extracted the following data from the included studies: general information (name of the first author, publication date), study population (age, sex, number of events, and sample size), designs of study, treatment options (type, dose, and duration), indicators related to adverse events and follow-up time.

### Quality evaluation

Two researchers (Y.Z. and J.L.) independently assessed the quality of the included studies. And the Newcastle-Ottawa Scale (NOS) was used to score the quality of the literature with cohort study. The scale has a total of 9 items in 3 broad categories: selectivity (4 items), comparability (2 items) and outcome (3 items). The overall quality was divided into three types: high quality (7–9 points), medium quality (4–6 points), and low quality (1–3 points). Scores ≥ 6 were considered to meet the inclusion criteria. Use the Cochrane bias risk assessment tools to evaluate the quality of the included randomized controlled trials (RCTs), and use the Review Manager 5.4.1 software to map the risk of bias graph. The evaluation items included randomized method, hidden assignment, blinded implementation, data integrity, selective reporting, and other bias, and each item was judged by “low risk of bias”, “uncertainty of bias” and “high risk of bias”. If there is any objection, the third researcher will help resolve it.

### Statistical methods

Review Manager 5.4.1 and Stata 15.1 were used to analyze the extracted data. Odds ratio (OR), 95% confidence interval (CI), and *p* value were used as analysis statistics for binary categorical variables. A random-effects meta-analysis model was used to combine treatment rates [25]. Heterogeneity was assessed using the Cochrane *Q* test and the *I*^2^ index by Review Manager 5.4.1 software. A series of stratified analyses were done to explore the origins of heterogeneity, in which the following factors were considered: study designs, ages, or follow-up time. The risk of publication bias was evaluated by visual inspection of a funnel plot when there were 10 trials or more, and further checked by the Begg's and Egger's tests. No publication bias was considered when *p* > 0.05.

## Results

### Literature screening process and results

**Fig. 1:**
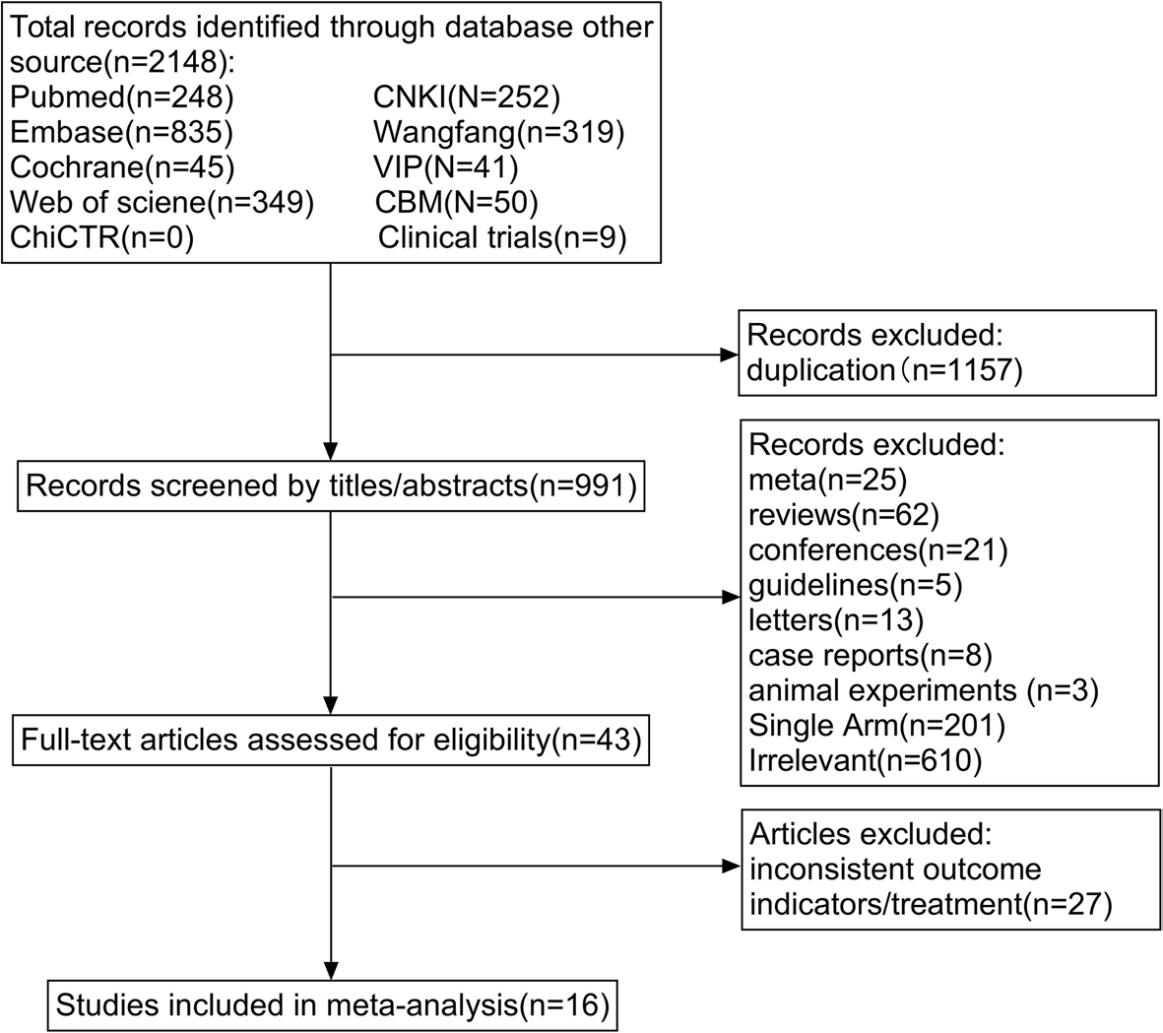
Flow diagram of the literature search

Two thousand one hundred forty-eight records were obtained from the preliminary search. One thousand one hundred fifty-seven duplicate records were excluded before the screening. Nine hundred forty-eight records were eliminated by reading titles/abstracts, and 43 records were included in the full-text evaluation. Twenty-seven articles were excluded because of inconsistent outcomes or treatment and 16 studies were finally selected. The retrieval process and results are shown in Fig. 1.

### Literature characteristics and quality evaluation

This meta-analysis included 16 studies [8, 26–40] with a total of 2148 patients. Three of the studies enrolled patients ≥ 18 years of age, one study only enrolled elderly patients, seven studies only enrolled children, and five studies did not set age limits. The general characteristics of the included studies are given in Table 1. Among the 16 included articles, the quality evaluation results of 13 cohort studies were shown in Supplementary Table S1, of which 4 studies had a NOS score of 8, 7 studies had a score of 7, and 2 study had a score of 6, all meeting the study criteria. The Cochrane risk of bias assessment of the three RCTs was low risk of bias and high quality. The results are given in Supplementary Figure S1.

**Table 1:** The general characteristics of the included studies

Author	Year	Sudy design	Types of patients/years old	Participants	Number of case		Intervention		Gender (male/female)/case		Median age (range)/year		Dose of EPAG		EPAG duration month	Median follow-up time (range) /month	
					EPAG+IST	IST	EPAG+IST	IST	EPAG+IST	IST	EPAG+IST	IST	Introduce	Maximum		EPAG+IST	IST
Assi [26]	2018	Cohort study	≥ 18	38	21	17	EPAG +hATG+CsA	hATG+CsA	11/10	8/9	60 (19–84)	53 (24–80)	50 mg/day	150 mg/day	≥ 6	21 (3-49)	
Chai [27]	2021	Cohort study	Elderly	35	24	11	EPAG +rATG+CsA	rATG+CsA	15/9	5/6	66.5 (61–81)	65 (61–72)	25 mg/day	75 mg/day	3 or 6	–	–
De Latour [33]	2022	RCT	Unlimited	197	96	101	EPAG +hATG+CsA	hATG+CsA	56/40	52/49	55 (16–77)	52 (15–81)	150 mg/day		6	23 (19–24)	24 (23–24)
Fang [28]	2021	Cohort study	< 18	57	18	39	EPAG +pATG+CsA	pATG+CsA	10/8	21/18	6.5 (1.2–12.5)	7 (1.6–13.5)	1 mg/kg/day	50 mg/day	6	31 (19–40)	69 (41–103)
Fang [36]	2023	RCT	Unlimited	90	45	45	EPAG +rATG+CsA	rATG+CsA	23/22	25/20	36 (15–60)	36 (15–59)	25 mg/day	75 mg/day	≥ 4	–	–
Goronkova [38]	2023	RCT	< 18	98	49	49	EPAG +hATG+CsA	hATG+CsA	35/14	30/19	10.5 (2–17.7)	8.7 (2.1–16.8)	2 mg/kg/day		3 or 6	26 (1–55)	3 or 6
Groarke [29]	2021	Cohort study	< 18	127	40	87	EPAG +hATG+CsA	hATG+CsA	23/17	51/36	13 (3–17)	11 (2–17)	≥ 12 years:150 mg/day 6–11 years:75 mg/day 2–5 years:25 mg/kg/day		≥ 3	47	80
Hu [37]	2022	Cohort study	Unlimited	111	37	74	EPAG +r/pATG+CsA	r/pATG+CsA	24/13	34/40	33 (10-68)	33 (10–68)	< 12 years:25 mg/day ≥ 12 years:50–75 mg/day	150mg/d	–	–	–
Jie [30]	2021	Cohort study	< 18	42	14	28	EPAG +rATG+CsA	rATG+CsA	8/6	14/15	7 (2–15.5)	8 (4–14)	≥ 6 years:75 mg/day 2–6 years:2.5 mg/kg/day		≥ 6	28 (25–32)	28.5 (1–88)
Jin [34]	2022	Cohort study	≥ 18	121	54	67	EPAG +rATG+CsA	rATG+CsA	28/26	30/37	39 (18–74)	40 (18–66)	25 mg/day	75 mg/day	–	14 (1–79)	16 (1–79)
Lesmana [31]	2021	Cohort study	< 18	25	9	16	EPAG +hATG+CsA	hATG+CsA	7/2	4/12	11 (4–18)	11.5 (1–17)	< 6 years:25 mg/day 6–18 years:50 mg/day	150 mg/day	6	15 (11–36)	86 (4–132)
Patel [35]	2022	Cohort study	Unlimited	280	178	102	EPAG +hATG+CsA	hATG+CsA	–	–	–	–	150 mg/day		3 or 6	48.7 (2.8–99.2)	87.8 (2.9–190.8)
Zaimoku [8]	2022	Cohort study	Unlimited	416	176	240	EPAG +hATG+CsA	hATG+CsA	87/89	141/99	32 (3–82)	30 (2-82)	> 11 years:150 mg/day 6–11 years:75 mg/day 2–5 years:2.5 mg/kg/day		6	–	–
Zhang [32]	2022	Cohort study	< 18	63	31	32	EPAG +rATG+CsA	rATG+CsA	16/15	16/16	12 (8–16)		≥ 27 kg:50 mg/day < 27 kg:1.5 mg/kg/day		3.33–22.30	–	–
Zhao [39]	2023	Cohort study	< 18	60	15	45	EPAG +r/pATG+CsA	r/pATG+CsA	10/5	26/19	13 (4–18)	13(7-17)	2.5 mg/kg/day	50 mg/day	0.1–51	19 (7–34)	74 (1–119)
Shinn [40]	2023	Cohort study	≥ 18	82	48	34	EPAG +hATG+CsA	hATG+CsA	25/23	18/16	54 (20–80)	39.5 (18–76)	150 mg/day		–	18 (0.8–70)	49 (0.4–93)

*IST* immunosuppressive therapy, *EPAG* eltrombopag, *RCT* randomized controlled trial, *hATG* horse antityhymocyte globulin, *rATG* rabbit antityhymocyte globulin, *pATG* pig antityhymocyte globulin, *CsA* cyclosporin A

### Results of meta-analysis

#### Overall response rate (ORR)

Fifteen studies compared the ORR difference between IST combined with EPAG and IST for SAA treatment.

**Fig. 2:**
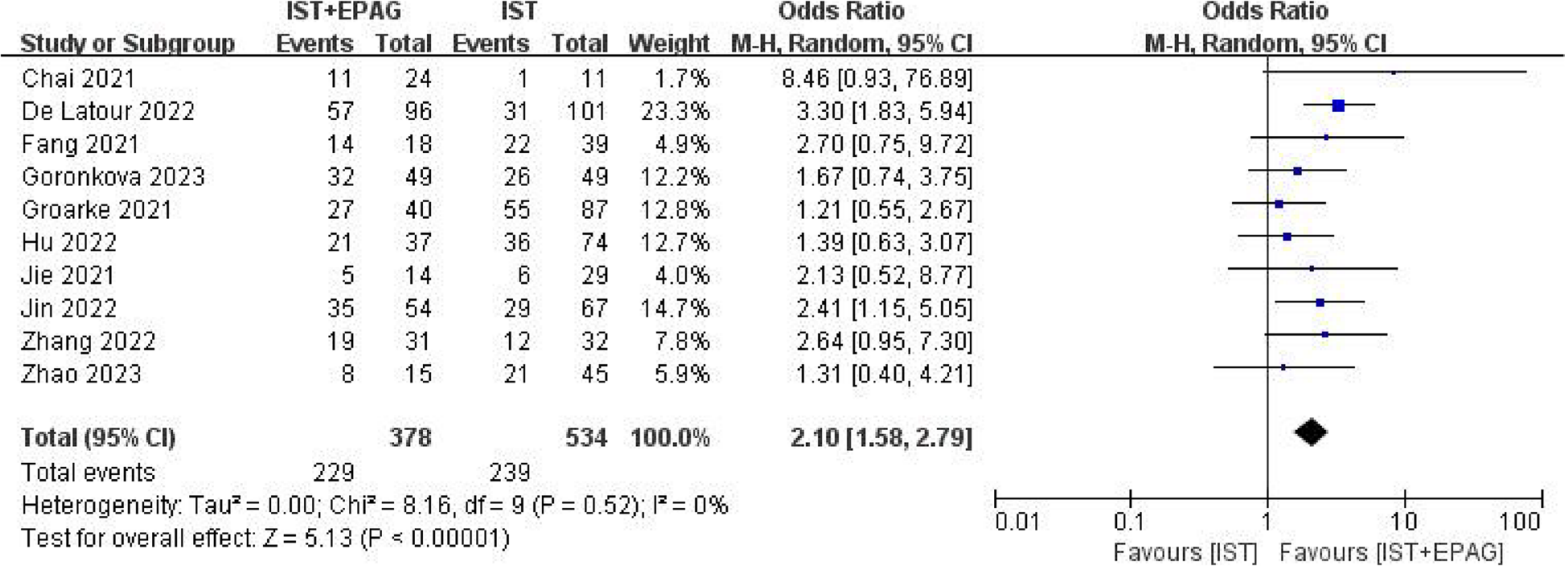
Overall response rate (ORR) at 3 months. EPAG: eltrombopag, IST: immunosuppressive therapy, CI: confidence interval

Ten out of fifteen studies described the ORR at 3 months. The results of the meta-analysis shown in Fig. 2, which indicated that IST combined with EPAG could improve the 3 months ORR of SAA patients (pooled OR = 2.10, 95% CI 1.58–2.79, *p* < 0.00001). There was no heterogeneity among these studies (*p* = 0.52, *I*^2^ = 0%).

**Fig. 3:**
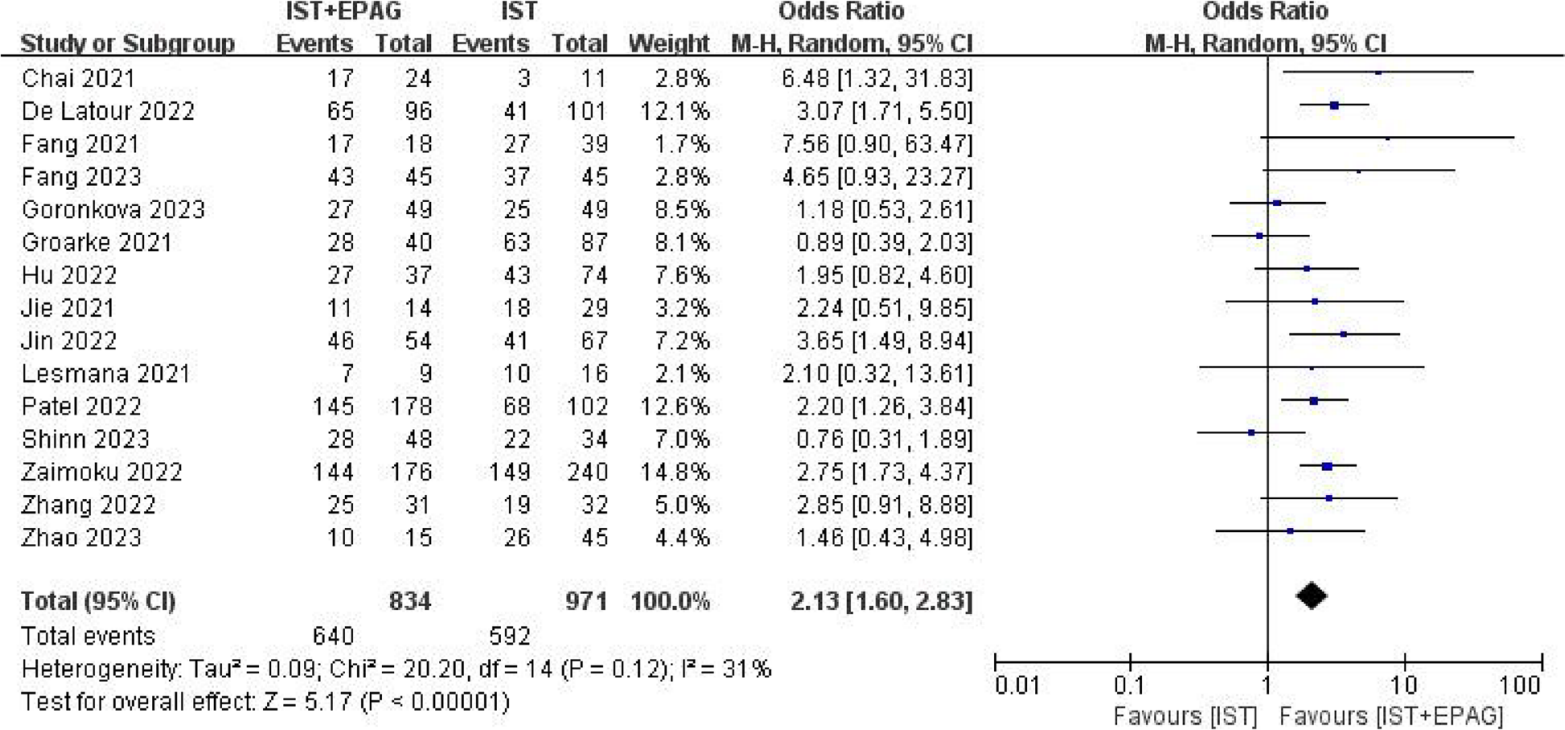
Overall response rate (ORR) at 6 months. EPAG: eltrombopag, IST: immunosuppressive therapy, CI: confidence interval

All studies described the ORR at 6 months. The results of meta-analysis showed in Fig. 3, which indicated that IST combined with EPAG could improve the 6 months ORR of SAA patients (pooled OR = 2.13, 95% CI 1.60–2.83, *p* < 0.00001). There was no heterogeneity among these studies (*p* = 0.12, *I*^2^ = 31%).

**Fig. 4:**
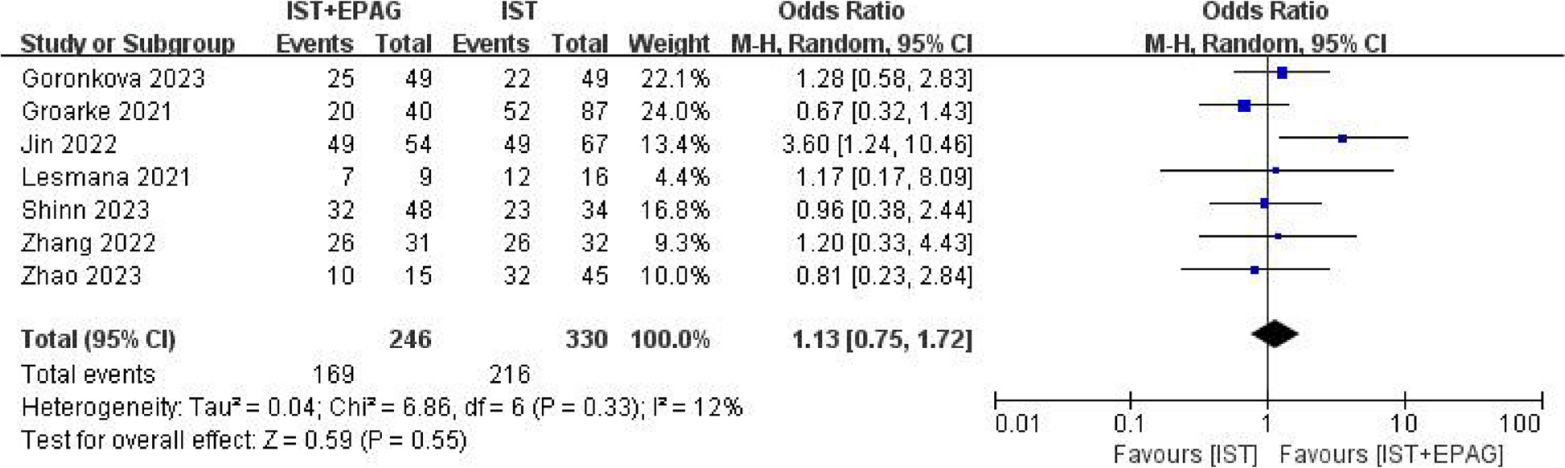
Overall response rate (ORR) at 12 months. EPAG: eltrombopag, IST: immunosuppressive therapy, CI: confidence interval

Seven out of 15 studies described the ORR at 12 months. The results of the meta-analysis shown in Fig. 4, which indicated that EPAG added to IST had no effect on 12 months ORR of SAA patients (pooled OR = 1.13, 95% CI 0.75–1.72, *p* = 0.55). There was no heterogeneity among these studies (*p* = 0.33, *I*^2^ = 12%).

#### Complete response rate (CRR)

Fourteen studies compared the CRR difference between IST combined with EPAG and IST for SAA treatment.

**Fig. 5:**
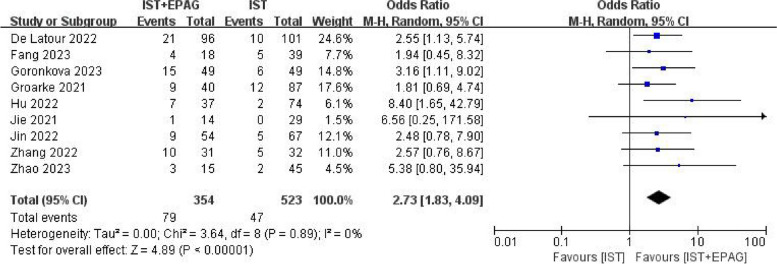
Complete response rate (CRR) at 3 months. EPAG: eltrombopag, IST: immunosuppressive therapy, CI: confidence interval

Nine out of 14 studies described the CRR at 3 months. The results of the meta-analysis shown in Fig. 5, indicated that IST combined with EPAG could improve the 3 months CRR of SAA patients (pooled OR = 2.73, 95% CI 1.83–4.09, *p* < 0.00001). There was no heterogeneity among these studies (*p* = 0.89, *I*^2^ = 0%).

**Fig. 6:**
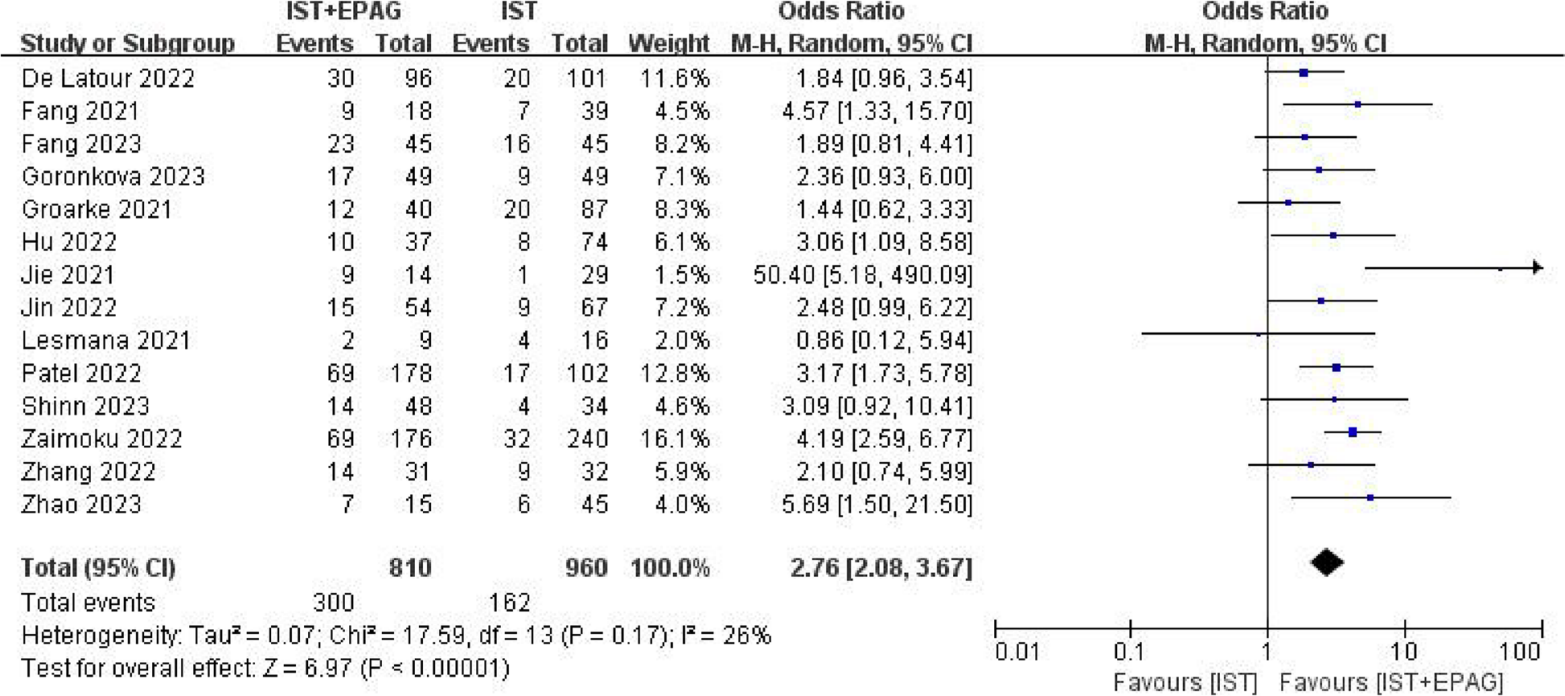
Complete response rate (CRR) at 6 months. EPAG: eltrombopag, IST: immunosuppressive therapy, CI: confidence interval

All studies described the CRR at 6 months. The results of meta-analysis showed in Fig. 6, which indicated that IST combined with EPAG could improve the 6 months CRR of SAA patients (pooled OR = 2.76, 95% CI 2.08–3.67, *p* < 0.00001). There was no heterogeneity among these studies (*p* = 0.17, *I*^2^ = 26%).

**Fig. 7:**
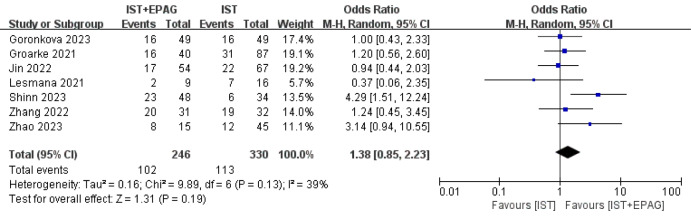
Complete response rate (CRR) at 12 months. EPAG: eltrombopag, IST: immunosuppressive therapy, CI: confidence interval

Seven out of 14 studies described the CRR at 12 months. The results of the meta-analysis shown in Fig. 7, indicated that IST combined with EPAG had no effect on 12 months CRR of SAA patients (pooled OR = 1.38, 95% CI 0.85–2.23, *p* = 0.19). There was no heterogeneity among these studies (*p* = 0.13, *I*^2^ = 39%).

#### Overall survival rate (OSR)

**Fig. 8:**
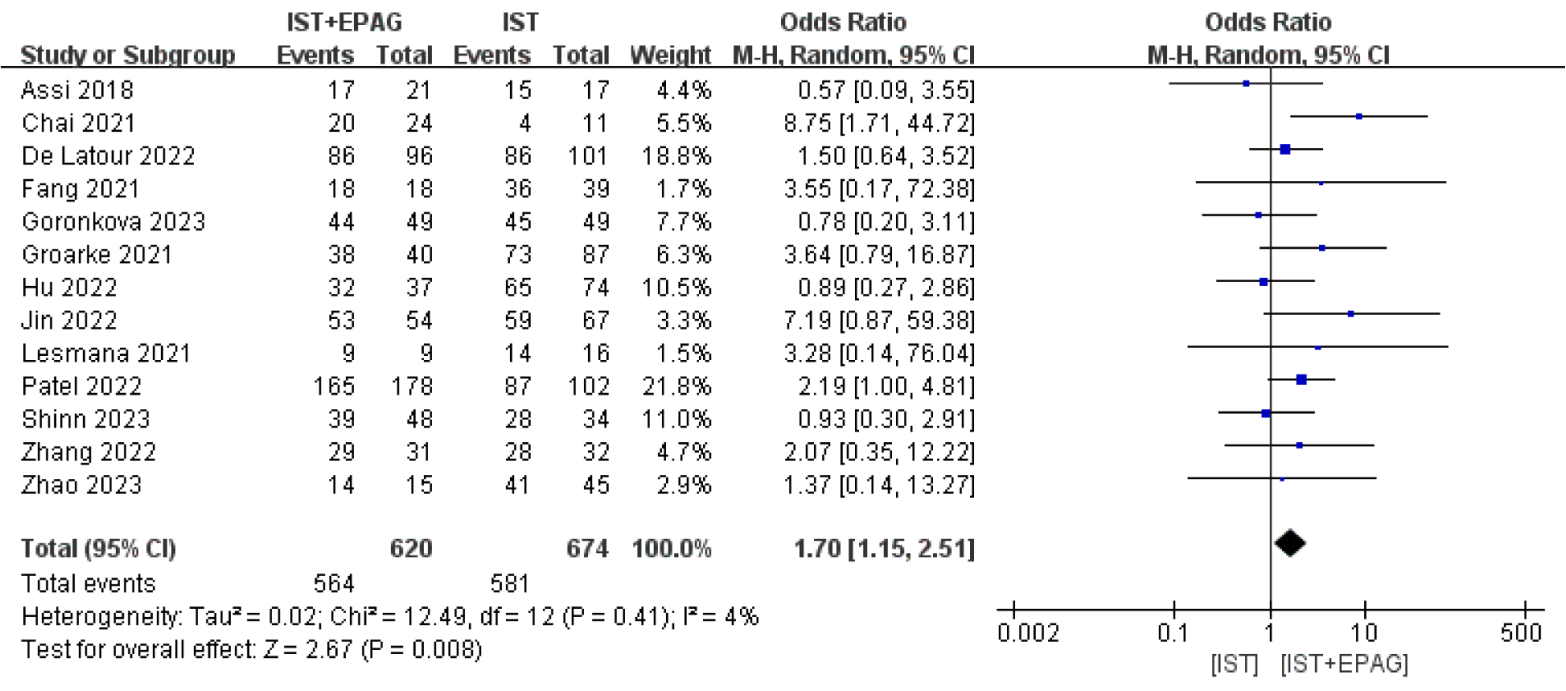
Overall survival rate (OSR). EPAG: eltrombopag, IST: immunosuppressive therapy, CI: confidence interval

Thirteen studies compared the difference in OSR between IST combined with EPAG and IST for the treatment of SAA patients. The results of the meta-analysis shown in Fig. 8, which indicated that IST combined with EPAG could improve the overall survival rate of SAA patients (pooled OR = 1.70, 95% CI 1.15–2.51, *p* = 0.008). There was no heterogeneity among these studies (*p* = 0.41, *I*^2^ = 4%).

#### Event-free survival rate (EFSR)

**Fig. 9:**
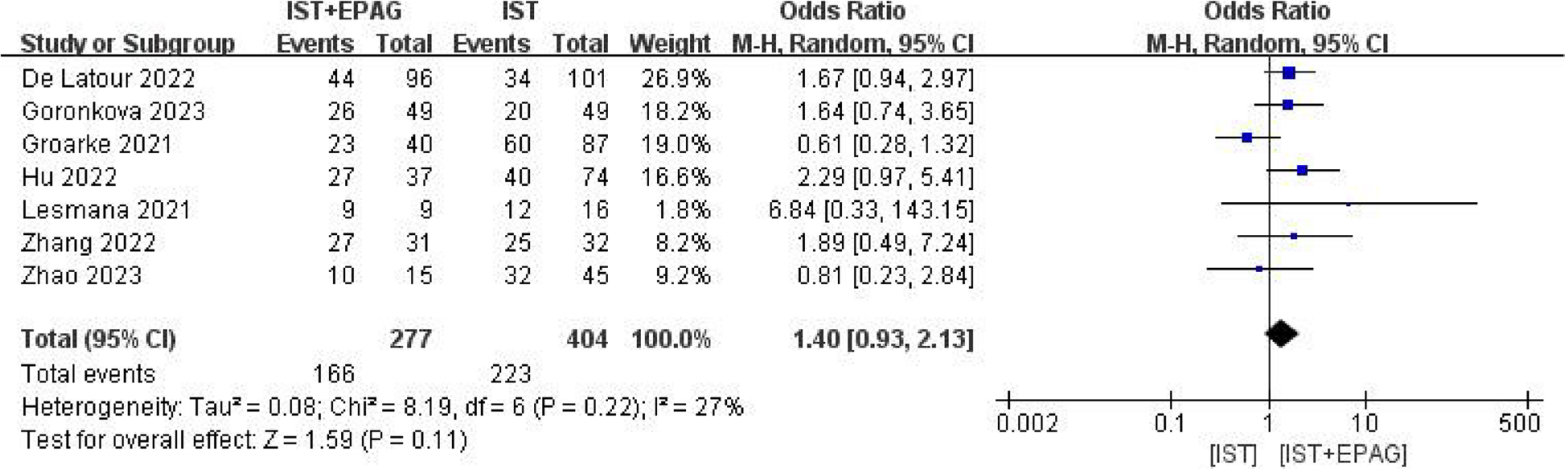
Event-free survival rate (EFSR). EPAG: eltrombopag, IST: immunosuppressive therapy, CI: confidence interval

Seven studies compared the difference in EFSR between IST combined with EPAG and IST for the treatment of SAA patients. The results of the meta-analysis shown in Fig. 9, which indicated that IST combined with EPAG had no effect on the event-free survival rate of SAA patients (pooled OR = 1.40, 95% CI 0.93–2.13, *p* = 0.11). There was no heterogeneity among these studies (*p* = 0.22, *I*^2^ = 27%).

#### Clone evolution rate

**Fig. 10:**
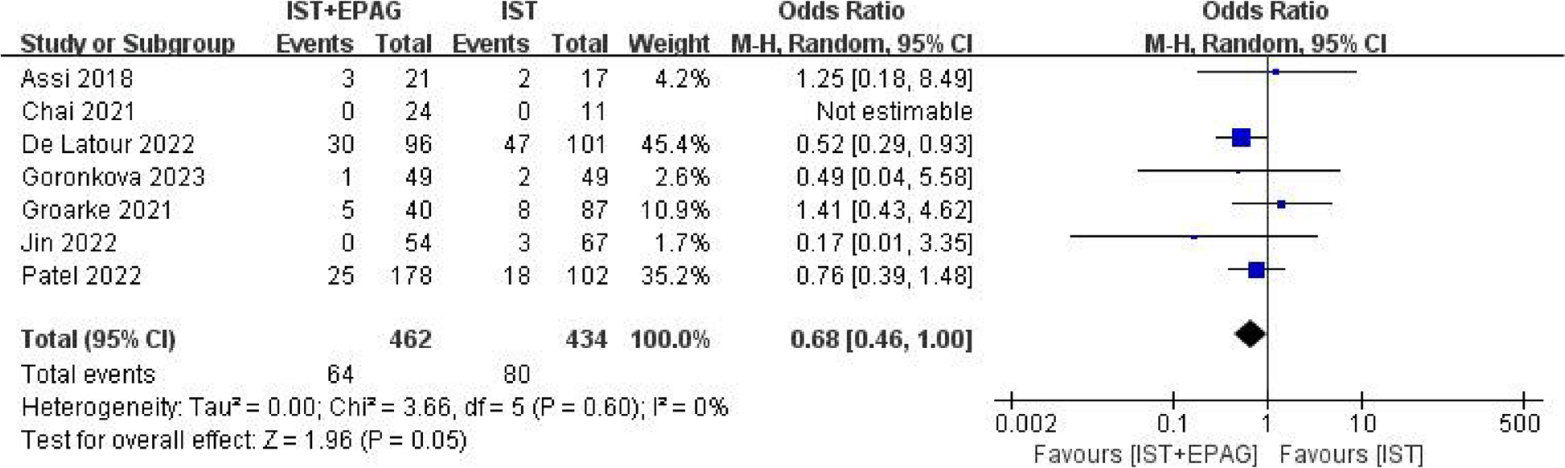
Clone evolution rate. EPAG: eltrombopag, IST: immunosuppressive therapy, CI: confidence interval

Seven studies compared the difference in clonal evolution rate between IST combined with EPAG and IST for the treatment of SAA patients. The results of the meta-analysis shown in Fig. 10, which indicated that IST combined with EPAG did not increase the incidence of clonal evolution rate of SAA patients (pooled OR = 0.68, 95% CI 0.46–1.00, *p* = 0.05). There was no heterogeneity among these studies (*p* = 0.60, *I*^2^ = 0%).

### Subgroup analysis

#### Subgroup analysis of different study designs

Using ORR and CRR as indexes, subgroup analysis was performed according to the study design (Table 2). The subgroup analysis results of different study designs indicated that whether in the cohort study subgroup or RCT subgroup, IST combined with EPAG could improve the ORR and CRR at 3 and 6 months of SAA patients, but had no effect on 12 months CRR of SAA patients. The *p* values for the interactions were all greater than 0.05, suggesting that ORR and CRR were not influenced by the study design.

**Table 2:** Subgroup analysis of different study designs

Outcomes	Subgroup	Number of included studies	IST+/IST	Pooled effects			*p* value for interaction	Heterogeneity	
				OR	95% CI	*p* value		*I*^2^/%	*p* value
ORR at 3 months	Cohort studies	8	233/384	1.86	[1.31, 2.65]	**0.0006**	0.297	0	0.64
	RCT	2	145/150	2.49	[1.29, 4.82]	**0.007**		44	0.18
ORR at 6 months	Cohort studies	12	644/776	2.08	[1.50, 2.89]	**< 0.0001**	0.815	30	0.15
	RCT	3	190/195	2.30	[1.07. 4.92]	**0.03**		54	0.11
ORR at 12 months	Cohort studies	6	197/281	1.12	[0.66, 1.89]	0.68	0.849	26	0.24
	RCT	1	49/49	1.28	[0.58, 2.83]	**–**		–	0.54
CRR at 3 months	Cohort studies	7	209/373	2.71	[1.62, 4.56]	**0.0002**	0.967	0	0.74
	RCT	2	145/150	2.76	[1.45, 5.25]	**0.002**		0	0.75
CRR at 6 months	Cohort studies	11	620/765	3.11	[2.20, 4.41]	**< 0.00001**	0.122	29	0.17
	RCT	3	190/195	1.97	[1.25, 3.09]	**0.003**		0	0.91
CRR at 12 months	Cohort studies	6	197/281	1.48	[0.83, 2.62]	0.18	0.620	46	0.10
	RCT	1	49/49	1.00	[0.43, 2.33]	–		–	1.00

Bold values indicate statistically significant values at *p* < 0.05

*IST* immunosuppressive therapy, *EPAG* eltrombopag, *OR* odds ratio, *CI* confidence interval, *ORR* overall response rate, *CRR* complete response rate, *RCT* randomized controlled trial

#### Subgroup analysis of different ages

Using ORR and CRR as indexes, subgroup analysis was also performed according to age (Table 3).

**Table 3:** Subgroup analysis of different ages

Outcomes	Subgroup	Number of included studies	EPAG+IST/IST	Pooled effects			*p* value for interaction	Heterogeneity	
				OR	95% CI	*p* value		*I*^2^/%	*p* value
ORR at 3m	< 18 years old	6	167/281	1.72	[1.14, 2.59]	**0.01**	0.239	0	0.82
	≥ 18 years old	2	78/78	2.90	[1.21, 6.99]	**0.02**		12	0.29
	Unlimited	2	133/175	2.23	[0.96, 5.20]	0.06		66	0.08
ORR at 6 months	< 18 years old	7	176/297	1.49	[0.97, 2.28]	0.07	0.071	0	0.44
	≥ 18 years old	3	126/112	2.39	[0.67, 8.46]	0.18		75	0.02
	Unlimited	5	532/562	2.61	[1.97, 3.46]	**< 0.00001**		0	0.80
ORR at 12 months	< 18 years old	5	144/229	0.94	[0.60, 1.48]	0.79	0.260	0	0.82
	≥ 18 years old	2	102/101	1.81	[0.49, 6.64]	0.37		70	0.07
	Unlimited	0	**–**	**–**	**–**	**–**		**–**	**–**
CRR at 3 months	< 18 years old	6	167/281	2.55	[1.50, 4.35]	**0.0006**	0.639	0	0.89
	≥ 18 years old	1	54/67	2.48	[0.78, 7.90]	–		**–**	0.12
	Unlimited	2	133/175	3.72	[1.25, 11.07]	**0.02**		39	0.20
CRR at 6 months	< 18 years old	7	176/297	2.92	[1.51, 5.67]	**0.002**	0.841	50	0.06
	≥ 18 years old	2	102/101	2.69	[1.29, 5.59]	**0.008**		0	0.78
	Unlimited	5	532/562	2.87	[2.04, 4.04]	**< 0.00001**		23	0.27
CRR at 12 months	< 18 years old	5	144/229	1.22	[0.77, 1.94]	0.39	0.546	4	0.38
	≥ 18 years old	2	102/101	1.92	[0.43, 8.52]	0.39		81	0.02
	Unlimited	0	–	–	–	–		–	–

Bold values indicate statistically significant values at *p* < 0.05

*IST* immunosuppressive therapy, *EPAG* eltrombopag, *OR* odds ratio, *CI* confidence interval, *ORR* overall response rate, *CRR* complete response rate

The results of < 18 years age subgroup analysis indicated that IST combined with EPAG could improve the ORR at 3 months and CRR at 3 and 6 months of SAA patients, but had no effect on 6 months and 12 months ORR and 12 months CRR.

The results of ≥ 18 years age subgroup analysis indicated that IST combined with EPAG could improve the ORR at 3 of SAA patients, and had no effect on 6 and 12 months, but could improve the CRR at 6 and 12 months of SAA patients. In addition, only 1 study met the inclusion criteria at 3 months CRR rendering the statistical analysis invalid.

The results of without age limit subgroup analysis indicated that IST combined with EPAG had no effect on 3 months ORR of SAA patients, but could improve the ORR at 6 months and CRR at 3 and 6 months of SAA patients. There were no eligible data for either ORR or CRR at 12 months.

The *p* values for the interactions were all greater than 0.05, suggesting that ORR and CRR were not influenced by the age of patients.

#### Subgroup analysis of different follow-up time

Using OSR and EFSR as indexes, subgroup analysis was also performed according to age (Table 4).

**Table 4:** Subgroup analysis of different follow-up time

Outcomes	Subgroup	Number of included studies	EPAG+IST/IST	Pooled effects			*p* value for interaction	Heterogeneity	
				OR	95% CI	*p* value		*I*^2^,%	*p* value
OSR	< 2 years	3	48/72	4.41	[1.30, 14.97]	**0.02**	0.135	0	0.42
	≥ 2 years	10	572/602	1.54	[1.03, 2.28]	**0.03**		0	0.52
EFSR	< 2 years	2	24/61	1.50	[0.22, 10.39]	0.68	0.787	41	0.19
	≥ 2 years	5	253/343	1.44	[0.91, 2.28]	0.12		37	0.17

Bold values indicate statistically significant values at *p* < 0.05

*IST* immunosuppressive therapy, *EPAG* eltrombopag, *OR* odds ratio, *CI* confidence interval, *OSR* overall survival rate, *EFSR* event-free survival rate

The subgroup analysis results of different follow-up times indicated that IST combined with EPAG could improve the OSR and EFSR of SAA patients in both < 2 years and ≥ 2 years. The *p* values for the interactions were all greater than 0.05, suggesting that OSR and EFSR were not influenced by follow-up time.

### Assessment of publication bias

Review Manager 5.4.1 was used to evaluate results of 3 months ORR, 6 months ORR, and OSR for publication bias, and funnel plots were drawn as shown in Supplementary Figures S2, S3, and S4. No evidence of asymmetry was shown. Then, we further carried out Begg’s and Egger’s test, and the results suggested that there was no publication bias in this study (3 months ORR: Begg’s test *p* = 0.721, Egger’s test *p* = 0.832; 6 months ORR: Begg’s test *p* = 0.274, Egger's test *p* = 0.676; OSR: Begg’s test *p* = 0.583, Egger’s test *p* = 0.361).

## Discussion

Lesmana et al.’s study [31] shows that IST combined with EPAG had no effect on ORR (100% vs 71%, *p* = 0.25; 100% vs 100%, *p* = 1) and CRR (29% vs 29%, *p* = 1; 29% vs 58%, *p* = 0.35) at 6 and 12 months. Jin et al.’s study [34] showed that the ORR (64%, 85%, 91%) at 3, 6, and 12 months in the IST combined with the EPAG group were higher than the IST group (44%, 61%, 73%) (*p* = 0.002, 0.028, 0.006, 0.031). While CRR was similar between the two groups (17% vs 7%, *p* = 0.069; 27% vs 14%, *p* = 0.11 and 32% vs 33%, *p* = 0.92). However, Hu’s study [37] showed that IST combined EPAG could improve patients’ ORR at 3 and 6 months, and significantly improve patients’ CRR at 3 and 6 months. Our meta-analysis showed that compared with IST, IST combined with EPAG could improve ORR and CRR at 3 months and 6 months, but there was no effect on the ORR and CRR at 12 months. This suggests that the addition of EPAG can enable SAA patients to obtain hematologic remission earlier and faster, reduce the dependence of patients on blood products, shorten the average hospital stay, reduce the economic burden of patients, and thus improve the quality of life of patients. Subgroup analysis based on different study types showed that in both the cohort study group and the RCT group, the addition of EPAG on the basis of IST could significantly improve the ORR and CRR of patients at 3 months and 6 months, while the ORR and CRR of patients at 12 months were not statistically significant between the two groups. The results of the subgroup analysis were consistent with the initial pooled results, suggesting that differences in study design were not the main source of heterogeneity. However, subgroup analysis based on different ages showed that in the subgroup of < 18 years old and ≥ 18 years old patients had similar results of ORR at 6 and 12 months between IST+EPAG group and IST group, while the CRR at 12 months was significantly higher than that in the IST group. ORR at 3 months in the IST+EPAG group was similar to that in the IST group in the unlimited age group. These were completely contrary to the results of previous meta-analyses. This difference between different age groups may be helpful for future protocols and decision-making regarding SAA treatment.

Studies have shown that the OSR and EFSR of SAA patients in the IST combined EPAG group were slightly higher, but not statistically significant, our meta-analysis showed that the addition of EPAG had a positive impact on the OSR and EFSR of patients, suggesting that the time and quality of hematological response may be predictors of long-term survival. Subgroup analyses based on different follow-up times showed that the addition of EPAG to IST increased OSR in SAA patients, but did not affect EFSR in SAA patients, in both the < 2-year group and the ≥ 2-year group. The results of the subgroup analysis were consistent with the previous pooled results, suggesting that differences in follow-up time were not the main source of heterogeneity. The *p* values for the interactions were all greater than 0.05, suggesting that the treatment effect was not influenced by subgroup characteristics.

The specific mechanism of clonal evolution in SAA patients is unclear. It is currently believed that abnormal immune responses initially eliminate abnormal cells at the expense of normal stem/progenitor cells, and over time, selective pressure leads to immune escape and pressure-selective cloning [41, 42]. At the same time, the intracellular telomerase activity of AA patients is reduced and telomere wear is accelerated compared with normal people, which leads to genomic instability, easy-to-develop acquired somatic mutations, and increases the risk of transformation into MDS/AML [43]. Studies have shown that 8–18% of patients treated with IST will develop clonal evolution [44–46]. TPO-RA stimulates in vivo expansion of surviving hematopoietic stem progenitors and accelerates telomere shortening [19], so it is very worrying whether the addition of EPAG will increase the risk of SAA clonal evolution. Patel et al.’s study [35] showed that the proportion of clonal evolution in patients was 15% during the 4-year follow-up period, and the median time of clonal evolution in the IST combined with the EPAG group was earlier. Similar results were also seen in other studies [47, 48]. However, other studies have found that the existing clonal evolution disappeared in SAA patients after EPAG treatment, and the mechanism remains unclear [49, 50]. Our meta-analysis showed that the incidence of clonal evolution in the IST combined with EPAG group was lower than that in the IST group, suggesting that EPAG does not increase the risk of clonal evolution in SAA patients, but may be related to the low incidence of clonal evolution. However, the median time from initiation of immunosuppressive therapy to occurrence of clonal evolution was 4–6 years [35]. At present, the follow-up time of most studies is still short, so it is necessary to follow up for a longer time, monitor the abnormalities of cell chromosomes and genetics in time, and regularly assess the risk of clonal evolution.

In addition, other common drug-related adverse events reported in patients treated with EPAG + IST were bilirubin increase (8%) and elevated liver enzymes (6%). Adverse events less related to EPAG included infection (25%), febrile neutropenia (23%), and renal damage (9%). Our statistical analysis showed no statistically significant differences between the IST+EPAG and IST groups in terms of adverse events, which were shown in Supplementary Table S2. It showed that the addition of EPGA did not increase the incidence of adverse events.

Our study has some limitations. First, due to the limited number of included studies and samples, the results of the meta-analysis may be affected. Second, the follow-up time of the included studies was relatively short, which may affect the observation of some outcome indicators. Therefore, needs more, larger, higher quality, and longer RCT clinical trials to further verify the results of our study.

## Conclusion

IST combined with EPAG can achieve earlier and faster hematologic remission with higher CRR. Although it had no effect on overall EFSR, it improved OSR and did not increase the incidence of clonal evolution and other adverse events.

### Supplementary Information

**Additional file 1:** **Supplementary Table S1.** Quality assessment of 13 cohort studies included in this Meta-Analysis. **Supplementary Table S2.** Results of meta-analysis of adverse events. **Supplementary Figure S1.** Quality assessment of three RCTs included in this Meta-Analysis. **Supplementary Figure S2.** Funnel plots of the included study in 3 months ORR ORR, and OSR. **Supplementary Figure S3.** Funnel plots of the included study in 6 months ORR. **Supplementary Figure S4.** Funnel plots of the included study in OSR.

## Data Availability

All data and material sources were included in this published article and electronic supplementary materials.
