# Supplementary material for: Efficacy and safety of immunosuppressive therapy combined with eltrombopag for severe aplastic anemia: a systematic review and meta-analysis
Source: Syst Rev. 2024 Apr 4;13:101. doi: 10.1186/s13643-024-02515-2 (PMC10993616; doi:10.1186/s13643-024-02515-2)

**Supplementary Materials**

**Journal: Systematic Reviews**

**Efficacy and safety of immunosuppressive therapy combined with eltrombopag for Severe**

**Aplastic Anemia: a systematic review and meta-analysis**

Running title： Efficacy and Safety of Eltrombopag for Severe Aplastic Anemia

Yan Zhang1, Jie Li2, Xi Li3, Qianshuang Geng4, Yuqin Xie4, Guoxiang Zhang4, Mingxia Wei4*,

Yanmei Ma1*

1. Department of Hematology, Heji Hospital Affiliated to Changzhi Medical college, Changzhi, 046000,

Shanxi, China

2. Department of Oncology and Hematology, Liuyang Hospital of Traditional Chinese Medicine,

Hunan University of Chinese Medicine, Changsha, 410300, Hunan, China

3. Department of Nephrology, Heping Hospital Affiliated to Changzhi Medical college, Changzhi,

046000, Shanxi, China

4. Department of Hematology, Heping Hospital Affiliated to Changzhi Medical college, Changzhi,

046000, Shanxi, China

Yan Zhang and Jie Li contribute equally to this article.

*Correspondence to: Mingxia Wei, Department of Hematology, Heping Hospital Affiliated to

Changzhi Medical college, Changzhi, 046000, Shanxi, China. Email: [zy15035865863@163.com](mailto:zy15035865863@163.com)

Yanmei Ma, Department of Hematology, Heji Hospital Affiliated to Changzhi Medical college,

Changzhi, 046000, Shanxi, China. Email: [514347026@qq.com](mailto:514347026@qq.com)

**Supplemental Table 1 Quality assessment of 13 cohort studies included in this Meta-Analysis**

**Supplemental Table 2 Results of meta-analysis of adverse events**

**Supplemental Fig. 1 Quality assessment of three RCTs included in this Meta-Analysis**

**Supplemental Fig. 2–4 Funnel plots of the included studies in 3-month ORR, 6-month ORR and OSR**

**Search strategies:**

Pubmed:

("anemia, aplastic"[MeSH Terms] OR ("aplastic anemias"[Title/Abstract] OR "aplastic anemia"[Title/Abstract] OR "aplastic anaemia"[Title/Abstract] OR "anaemia aplastic"[Title/Abstract] OR "aplastic anaemias"[Title/Abstract] OR "anemia hypoplastic"[Title/Abstract] OR "hypoplastic anemia"[Title/Abstract] OR "hypoplastic anemias"[Title/Abstract])) AND ("Eltrombopag"[Title/Abstract] OR "Eltrombopag"[Supplementary Concept] OR "SB-497-115"[Title/Abstract] OR "SB-497-115"[Title/Abstract] OR "SB497115"[Title/Abstract] OR "Promacta"[Title/Abstract] OR "Revolade"[Title/Abstract]) 248

Embase:

| #7 | #3 AND #6 | 835 |
| --- | --- | --- |
| #6 | #4 OR #5 | 3,923 |
| #5 | promacta' OR 'ddl-701' OR 'sb-497 115' OR 'sb497115' OR 'sb-497-115' OR 'revolade' | 287 |
| #4 | eltrombopag'/exp | 3,919 |
| #3 | #1 OR #2 | 62,386 |
| #2 | aplastic anemias':ab,ti OR 'aplastic anemia':ab,ti OR 'aplastic anaemia':ab,ti OR 'anaemia, aplastic':ab,ti OR 'aplastic anaemias':ab,ti OR 'anemia, hypoplastic':ab,ti OR 'hypoplastic anemia':ab,ti OR 'hypoplastic anemias':ab,ti | 17,305 |
| #1 | aplastic anemia'/exp | 60,493 |

Cochrane library:

| #1 | MeSH descriptor: [Anemia, Aplastic] explode all trees | 266 |
| --- | --- | --- |
| #2 | (Aplastic Anemias):ti,ab,kw OR (Aplastic Anemia):ti,ab,kw OR (Aplastic Anaemia):ti,ab,kw OR (Anaemia, Aplastic):ti,ab,kw OR (Aplastic Anaemias):ti,ab,kw OR (Anemia, Hypoplastic):ti,ab,kw OR (Hypoplastic Anemia):ti,ab,kw OR (Hypoplastic Anemias):ti,ab,kw | 698 |
| #3 | #1OR#2 | 696 |
| #4 | (eltrombopag):ti,ab,kw OR (Promacta):ti,ab,kw OR (SB-497 115):ti,ab,kw OR (SB497115):ti,ab,kw OR (DDL-701):ti,ab,kw OR (Revolade):ti,ab,kw | 345 |
| #5 | #3AND#4 | 45 |

Web of science:

| 3 | **(#1) AND #2** | **349** |
| --- | --- | --- |
| 2 | **Eltrombopag** (Topic) or **Promacta** (Topic) or **Revolade** (Topic) or **SB497115** (Topic) or **SB-497 115** (Topic) or **SB-497-115** (Topic) or **DDL-701** (Topic) | 1902 |
| 1 | **Anemia, Aplastic** (Topic) or **Aplastic Anemias** (Topic) or **Aplastic Anemia** (Topic) or **Aplastic Anaemia** (Topic) or **Anaemia, Aplastic** (Topic) or **Aplastic Anaemias** (Topic) or **Anemia, Hypoplastic** (Topic) or **Hypoplastic Anemia** (Topic) or **Hypoplastic Anemias** (Topic) | 14185 |

VIP（维普）：

(((((((题名或关键词=再生障碍性贫血 OR 题名或关键词=anemia aplastic) OR 题名或关键词=aplastic anemia) OR 题名或关键词=再障) OR 题名或关键词=贫血, 再生障碍性) OR 题名或关键词=再生不良性贫血) OR 题名或关键词=贫血，再生不良性) AND ((((题名或关键词=艾曲泊帕 OR 题名或关键词=艾曲波帕) OR 题名或关键词=Eltrombopag) OR 题名或关键词=Promacta) OR 题名或关键词=Revolade)) 41

Wanfang（万方）：

(((((((主题=艾曲泊帕) OR 主题=艾曲波帕) OR 主题=Eltrombopag) OR 主题=Revolade) OR 主题=瑞弗兰)) OR ((((主题=SB-497 115) OR 主题=SB-497-115) OR 主题=SB497115))) AND ((((((主题=再生障碍性贫血 ) OR 主题=再障) OR 主题=再生不良性贫血) OR 主题=(anemia aplastic)) OR 主题=(aplastic anemia))) 319

CNKI（知网）：

(主题：再生障碍性贫血+ anemia aplastic + aplastic anemia+再障+再生不良性贫血+贫血，再生不良性)AND（主题： 艾曲泊帕 +艾曲波帕 +Eltro mbopag + Promacta + (SB-497 115) + (SB-497-115) + SB497115 + Revol ade+ 瑞弗兰)+中英文扩展 252

CBM（中国生物医学文献服务系统）：

| 5 | (#4) AND (#3) | | 50 |
| --- | --- | --- | --- |
| 4 | 艾曲泊帕[常用字段:智能] OR "艾曲波帕"[常用字段:智能] OR "Eltrombopag"[常用字段:智能] OR "Promacta"[常用字段:智能] OR "SB-497"[常用字段:智能] AND "115"[常用字段:智能] OR "SB-497-115"[常用字段:智能] OR "SB497115"[常用字段:智能] OR "Revolade"[常用字段:智能] OR "瑞弗兰"[常用字段:智能] | | 217 |
| 3 | (#2) OR (#1) | 11747 | |
| 2 | 再障[常用字段:智能] OR "anemia"[常用字段:智能] AND "aplastic"[常用字段:智能] OR "aplastic"[常用字段:智能] AND "anemia"[常用字段:智能] OR "再生不良性贫血"[常用字段:智能] OR "贫血,"[常用字段:智能] AND "再生不良性"[常用字段:智能] | 11747 | |
| 1 | 贫血, 再生障碍性[不加权:扩展] | | 9009 |

ChiCTR（中国临床试验中心）：

| 注册题目： | 再生障碍性贫血 |
| --- | --- |
| 干预措施： | 艾曲泊帕 |
| 招募研究对象情况： | 结束 |
| 获伦理委员会批准： | 是/Yes |
| 其余选项均未进行特殊限制 |  |
| 结果： | 0 |

Clinical trials：

| Condition/disease | Anemia，Aplastic |
| --- | --- |
| Intervention/treatment | Eltrombopag/Revolade |
| Study Results： | with results |
| The rest are not subject to special restrictions |  |
| Search Results | 9 |

**Supplemental Table 1 Quality assessment of 13 cohort studies included in this Meta-Analysis**

| **Studies** | **Selection** | **Comparability** | **Outcome** | **score** |
| --- | --- | --- | --- | --- |
| Assi 2018 | ★★★★ | ★ | ★★★ | **8** |
| Chai 2021 | ★★★★ | ★ | ★★★ | **8** |
| Fang 2021 | ★★★ | ★ | ★★★ | **7** |
| Groarke 2021 | ★★★★ | ★ | ★★★ | **8** |
| Lesmana 2021 | ★★★ | **–** | ★★★ | **6** |
| Jie 2021 | ★★★ | **–** | ★★★ | **6** |
| Hu 2022 | ★★★ | ★ | ★★★ | **7** |
| Zhang 2022 | ★★★ | ★ | ★★★ | **7** |
| Jin 2022 | ★★★ | ★ | ★★★ | **7** |
| Patel 2022 | ★★★★ | ★ | ★★★ | **8** |
| Zaimoku 2022 | ★★★ | ★ | ★★★ | **7** |
| Zhao 2023 | ★★★ | ★ | ★★★ | **7** |
| Shine 2023 | ★★★ | ★ | ★★★ | **7** |

**Supplemental Table 2 Results of meta-analysis of adverse events**

| Outcomes | Number of included studies | EPAG+IST /IST | Pooled effects | | | Heterogeneity | | |
| --- | --- | --- | --- | --- | --- | --- | --- | --- |
|  |  |  | OR | 95% CI | *p* value | | *I*^2^,% | *p* value |
| Bilirubin increase | 4 | 166/200 | 0.93 | [0.15, 1.72] | 0.83 | | 84 | 0.0003 |
| Elevated liver enzymes | 2 | 99/112 | 1.23 | [0.42, 3.59] | 0.70 | | 0 | 0.55 |
| Infection | 5 | 164/181 | 0.64 | [0.38, 1.10] | 0.11 | | 12 | 0.33 |
| Febrile neutropenia | 3 | 79/82 | 0.84 | [0.40, 1.76] | 0.64 | | 0 | 0.80 |
| Renal damage | 5 | 229/250 | 1.02 | [0.29, 3.59] | 0.97 | | 63 | 0.03 |

IST: immunosuppressive therapy, EPAG: eltrombopag, OR: odds ratio, CI: confidence interval

**Supplemental Fig. 1 Quality assessment of three RCTs included in this Meta-Analysis**


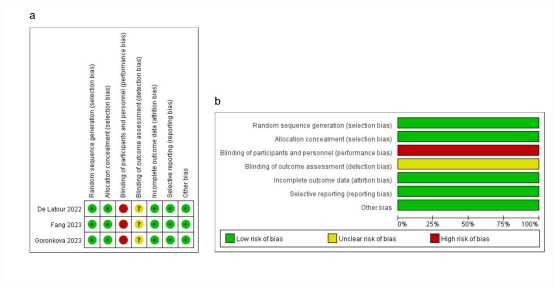


1. Risk of bias summary; b. Risk of bias graph

**Supplemental Fig. 2 Funnel plots of the included study in 3 months ORR**


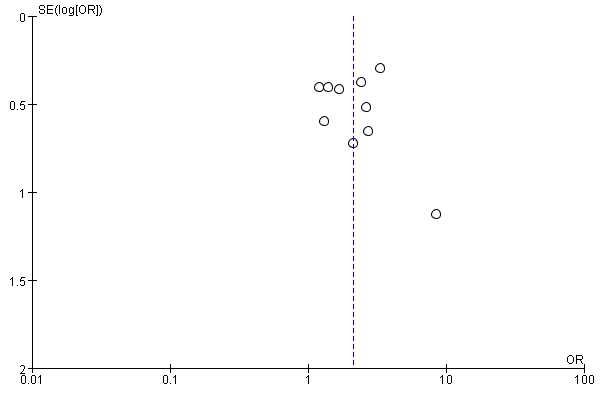


**Supplemental Fig. 3 Funnel plots of the included study in 6 months ORR**


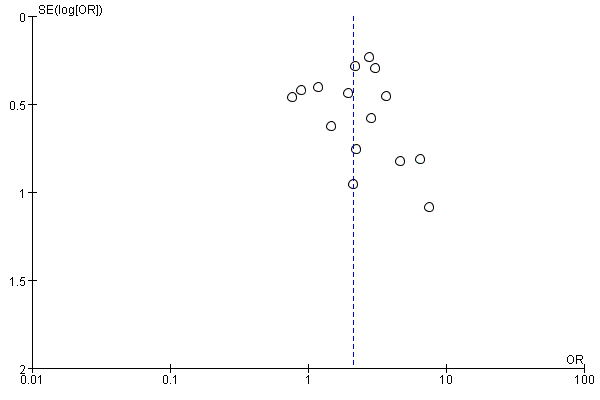


**Supplemental Fig. 4 Funnel plots of the included study in OSR**


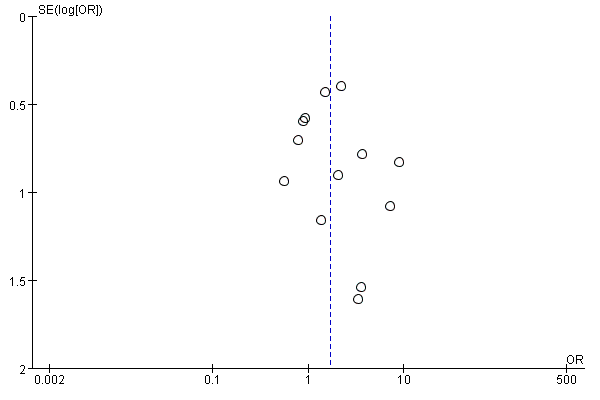

Supplement: Supplementary file 1 — Additional file 1: Supplementary Table S1. Quality assessment of 13 cohort studies included in this Meta-Analysis. Supplementary Table S2. Results of meta-analysis of adverse events. Supplementary Figure S1. Quality assessment of three RCTs included in this Meta-Analysis. Supplementary Figure S2. Funnel plots of the included study in 3 months ORR ORR, and OSR. Supplementary Figure S3. Funnel plots of the included study in 6 months ORR. Supplementary Figure S4. Funnel plots of the included study in OSR. [file 13643_2024_2515_MOESM1_ESM.docx]
